# Our experience in three coronary patients with heparin induced thrombocytopenia type II, platelets

**DOI:** 10.1186/1749-8090-8-S1-P77

**Published:** 2013-09-11

**Authors:** A Mikić, M Velinović, M Vraneš, R Karan, N Savić, B Nikolić, V Jovičić, M Čubrilo, N Aleksić, M Šamanović

**Affiliations:** 1 Clinic for Cardiac Surgery, Clinical Center of Serbia, Belgrade, Serbia; 2 Center for Anesthesiology, Clinic for Cardiac Surgery, Clinical Center of Serbia, Belgrade, Serbia; 3 Dpt of Transfusiology, Vascular Surgery Clinic, Clinical Center of Serbia, Belgrade, Serbia

## Background

Heparin induced thrombocytopenia type II (HIT type II) is well defined thrombophylic and immunological complication due to heparin therapy, which occurs in 1-3% treated patients. In around 50% of the patients with HIT type II have developed serious thrombotic arterial and venous complications. It has a special place in cardiac surgery, regarding standard use of heparin. In patients with HIT type II is contraindicated continuation of heparin therapy including use of low molecular heparin. Alternative drugs should be used as danaparoid sodium, lepirudin, argatroban.

## Methods

Coronary revascularization was done in three patients and mitral valve repair was done in one patient. First was male, 50 years old, who has suffered three myocardial infarctions (MI) previously and has developed left ventricular low ejection fraction of 25%. He had three vessels disease. We performed revascularization off pump (one arterial and one vein graft) using danaparoid sodium as alternative anticoagulation. Second patient was male, 65 years old, who previously suffered two MI and had three vessels disease. We did a coronary revascularization on pump (three vein grafts) using danaparoud sodium. Third coronary patient was male, 46 years old with two previous MI and three vessels disease. We performed revascularization off pump (two arterial and one vein graft) using danaparoid sodium, as alternative anticoagulation.

## Results

In all patients, during operation and postoperatively we had no complications, except in second patient who had superficial wound infection.

## Conclusion

The diagnosis and treatment of HIT type II needs to be considered on an individual basis, because of the variable presentation and severity of the syndrome. Special attention should be placed on platelet count in patients who received heparin therapy. In cardiac surgery, we need a better screening test to discovery the HIT type II patients who are previously treated with heparin.

